# Differentiation of advanced generation mutant wheat lines: Conventional techniques versus Raman spectroscopy

**DOI:** 10.3389/fpls.2023.1116876

**Published:** 2023-02-23

**Authors:** Ayse SEN, Ibrahim Kecoglu, Muhammad Ahmed, Ugur Parlatan, Mehmet Burcin Unlu

**Affiliations:** ^1^ Department of Biology, Faculty of Science, Istanbul University, Istanbul, Türkiye; ^2^ Department of Physics, Bogazici University, Istanbul, Türkiye; ^3^ Graduate School of Engineering and Science, Istanbul University, Istanbul, Türkiye; ^4^ Faculty of Engineering, Hokkaido University, Sapporo, Hokkaido, Japan; ^5^ Global Center for Biomedical Science and Engineering Quantum Medical Science and Engineering (GI-CoRE Cooperating Hub), Faculty of Medicine, Hokkaido University, Sapporo, Japan

**Keywords:** antioxidant enzymes, chlorophyll contents, TBARS content, wheat mutant lines, qPCR, proline, Raman spectroscopy, salinity stress

## Abstract

This research aimed to assess the feasibility of utilizing Raman spectroscopy in plant breeding programs. For this purpose, the evaluation of the mutant populations set up the application of 4 mM NaN_3_ to the somatic embryos obtained from mature wheat (*Triticum aestivum* L. Adana-99 cv.) embryos. Advanced wheat mutant lines, which were brought up to the seventh generation with salt stress tolerance by following *in vitro* and *in vivo* environments constructed by mutated populations, were evaluated using conventional techniques [measurement of antioxidant enzyme activities (SOD, CAT, and POX), total chlorophyll, TBARS, and proline contents; measurement of the concentration of Na^+^ and K^+^ ions; and evaluation of gene expression by qPCR (*Ta*HKT2;1, *Ta*HKT1;5, *Ta*SOS1, *Ta*Na^+^/H^+^ vacuolar antiporter, *Ta*V-PPase, *Ta*V-ATPase, and *Ta*P5CS)] and Raman spectroscopy. In this research, no significant difference was found in the increase of SOD, CAT, and POX antioxidant enzyme activities between the salt-treated and untreated experimental groups of the commercial cultivar, while there was a statistically significant increase in salt-treated advanced generation mutant lines as compared to control and the salt-treated commercial cultivar. Proline showed a statistically significant increase in all experimental groups compared to the untreated commercial cultivar. The degradation in the amount of chlorophyll was lower in the salt-treated advanced generation mutant lines than in the salt-treated commercial cultivar. According to gene expression studies, there were statistical differences at various levels in terms of Na^+^ and/or K^+^ uptake from soil to plant (T*a*HKT2;1, *Ta*HKT1;5, and *Ta*SOS1), and Na^+^ compartmentalizes into the cell vacuole (*Ta*Na^+^/H^+^ vacuolar antiporter, *Ta* vacuolar pyrophosphatase, and *Ta* vacuola*r* H^+^-ATPase). The expression activity of *Ta*P5CS, which is responsible for the transcription of proline, is similar to the content of proline in the current study. As a result of Raman spectroscopy, the differences in peaks represent the protein-related bands in mutant lines having a general decreasing trend in intensity when compared to the commercial cultivar. Amide-I (1,630 and 1,668 cm^−1^), Histidine, Lysine, Arginine, and Leucine bands (823, 849, 1,241, 1,443, and 1,582 cm^−1^) showed decreasing wavenumbers. Beta-carotene peaks at 1,153 and 1,519 cm^−1^ showed increasing trends when the normalized Raman intensities of the mutant lines were compared.

## 1 Introduction

*Triticum aestivum* L., commonly known as wheat, is an essential crop that plays a vital role in fulfilling the dietary requirements of the expanding human population around the world, providing approximately 20% of our energy needs (calories) and 25% of our protein intake. Soil with high levels of salinity can be detrimental for many crops, resulting in decreased growth and yield due to its harmful impact (Shrivastava and Kumar, 2015), threatening sustainability in agricultural production and world food security. While temporary measures can help mitigate the negative impact of soil salinity on plants, implementing these solutions is not always straightforward. To minimize yield loss due to salt stress, it is necessary to increase the tolerance of the affected crops to salt. This can often be achieved through breeding techniques that improve the crops in question. Advanced breeding techniques including genome editing methodologies and transgenic techniques are currently seen as promising approaches for increasing salt tolerance in crops; however, the use of these new technologies is limited due to the fact that wheat is an allohexaploid plant with a large genome and is relatively non-responsive in the tissue culture (Borisjuk et al., 2019; Chen et al., 2019). As a result, traditional breeding techniques are still utilized by wheat breeders. Using mutagenic agents to induce mutations in crops, followed by a selective breeding strategy, can be an effective way to generate crops with improved traits. Chemical mutagens, such as sodium azide (NaN_3_), are commonly used to create mutations in crops. These mutagens often lead to base pair mutations, particularly GC : AT, which can result in changes to the amino acid composition of proteins and alter their function (Dubey et al., 2017). As a result of the mutations induced by mutagens, plants can exhibit a wide range of variations in morphological and yield-related characteristics compared to normal plants. Scientists have demonstrated the role of induced mutations in increasing the genetic variability for agronomic traits in various crop plants (Chen et al., 2019). One measure of the success of this approach is the more than 3,390 mutant plant varieties that have been registered in the FAO/IAEA (Food and Agriculture Organization/International Atomic Energy Agency)’s mutant database (https://www.iaea.org/resources/databases/mutant-varieties-database).

Salt stress initiates many physiological and biochemical responses in mutant plants. Although there are conventional techniques currently used to evaluate these responses, modern techniques such as Raman spectroscopy (RS), as an alternative, have been tried to be introduced to the world of scientists recently (Payne and Kurouski, 2021). Among the conventional techniques, *in vitro* biochemical techniques based on a UV spectrophotometer, for example, measurement of antioxidant enzyme activities, total chlorophyll content and thiobarbituric acid-reactive substances (TBARS) as a membrane damage index are frequently used. In addition, examining the transcription profiles of stress-related genes analyzed using the quantitative polymerase chain reaction (qPCR) technique is also among the conventional techniques we use today.

Salt stress can cause oxidative stress in plants, resulting in greater production of reactive oxygen species (ROS) including singlet oxygen, hydrogen peroxide, hydroxyl radical, and superoxide. The formation of ROS in response to salinity can cause oxidative damage to different cellular components, including proteins, lipids, and DNA, disrupting important cellular functions in plants, which, in turn, can hinder crucial cellular functions in plants. The antioxidant metabolism, consisting of enzymatic and non-enzymatic antioxidant agents, plays a vital role in neutralizing ROS caused by salt stress. Tolerance to salinity is positively correlated with the antioxidant enzymes’ activity, for example, catalase (CAT: EC 1.11.1.6), superoxide dismutase (SOD : EC 1.15.1.1), and guaiacol peroxidase (POX: EC 1.11.1.7), and with the buildup of non-enzymatic antioxidant compounds (Hanin et al., 2016). Sajid and Aftab (2014) found that the levels of SOD, POX, and CAT enzymes were higher in salt-tolerant potato lines grown in a saline environment *in vitro* compared to the control group.

Proline compatible solutes (or compatible osmolytes) are small organic molecules that play a part to protect the structure and maintaining osmotic balance within cells. The accumulation of proline, a type of compatible osmolyte, is a common response to salinity stress and can help alleviate the negative effects of this stress. In addition to providing tolerance to salinity stress, proline accumulation during stress can also assist as a source of organic nitrogen for the plant during recovery from stress (Gupta and Huang, 2013). According to Kumar et al. (2017), the Kharchia-65 cultivar was the most salt-tolerant among the cultivars studied, likely due to its better membrane stability, higher levels of antioxidants, higher content of chlorophyll, higher osmolyte accumulation, and a higher K^+^/Na^+^ ratio under stress conditions. These characteristics may contribute to the plant’s ability to tolerate salt stress.

One of the major negative impacts of salt stress is the buildup of sodium and chloride ions in the tissues of plants grown in soils with high NaCl concentrations. The entry of sodium and chloride ions into plant cells can cause high imbalance of ions and excessive uptake can lead to disorders in physiology. High levels of Na^+^ can inhibit the uptake of potassium (K^+^), an important element for the development and growth of plant, ultimately leading to reduced yield and possibly even plant death. Maintaining ion homeostasis within plant cells is an important strategy for plants to tolerate stress conditions. High-Affinity Potassium Transporters (HKTs), the Sodium/Hydrogen Exchanger (SOS1), the Sodium/Hydrogen Antiporter (NHX), the Vacuolar Hydrogen ion-ATPase (V-ATPase), and the Vacuolar Hydrogen ion-pyrophosphatase (V-PPase) are all involved in regulating the uptake and compartmentalization of ions in plants. Typically, from the soil to the root, Na^+^ can pass passively, or HKT-type transporter can work. The HKT family of proteins is divided into two smaller groups: HKT1 and HKT2. With a preference for Na^+^, HKT1 is a carrier of K^+^/Na^+^ ions; on the other hand, HKT2 is permeable to both K^+^ and Na^+^. SOS1 (Salt Overly Sensitive) is alternative mechanism that regulates Na ion transport in plants. Contrasting to HKT1, SOS1 entries sodium ions from the cortex cells at the interface of root-soil (Feki et al., 2014). The tonoplast Na^+^/H^+^ antiporter, the V-ATPase (i.e., vacuolar H^+^-ATPase), and the V-PPase (i.e., vacuolar H^+^-pyrophosphatase) are believed to help maintain low levels of cytoplasmic sodium (Na^+^) in plant cells through their regulation of the transport of Na+ into the vacuole (V-ATPase). Salt stress caused an increase in the levels of transcripts (gene expressions) of *Hv*P1, *Hv*P10, *Hv*VHA-A, and *Hv*NHX1 in transgenic barley roots. Additionally, osmotic stress (a stress caused by an imbalance in the concentration of water and solutes within a cell) also leads to a rise in the levels of transcripts of *Hv*P1 and *Hv*NHX1 in transgenic barley roots (Fukuda et al., 2004). Increasing the amount of expression of TNHX1 and TVP1 genes in wheat plants enhances their ability to tolerate salt stress in *Arabidopsis* (Brini et al., 2007). The *Arabidopsis* HC-PPase (AtAVP1) gene has also been observed to improve stress tolerance in a crop plant. According to Schilling et al. (2013), barley plants that have been genetically modified to overexpress the *At*AVP1 gene are more resistant to salinity in greenhouse conditions. These transgenic plants also demonstrated an increase in shoot biomass production and grain yield in saline field conditions.

Conventional techniques (measurement of antioxidant enzyme activities, chlorophyll content, TBARS content, gene expression analysis, etc.) that we use today in investigating the biochemical, physiological, and molecular responses that occur in plants under stress conditions are more relevant, but are harm the plant, increase cost, labor and time loss. Considering the rapid increase in the world’s population, scientists have to rapidly introduce new high-output techniques to turn disadvantages such as time, cost, and labor loss into advantages in order to contribute to shortening the plant breeding process in order to meet the growing demand caused by the increasing population. RS is one of the vibrational spectroscopic methods, and it is an *in situ* technique that has been rapidly used in various fields such as basic biological research, agricultural research, and cancer research in a shorter time, with less labor and cost and without harming the plant (Farber et al., 2020).

In this study, the evaluation of the mutant population set up by the application of 4 mM sodium azide to the somatic embryos obtained from mature wheat embryos and the evaluation of the advanced generation wheat mutants, which were brought up to the seventh generation with salt stress tolerance by following *in vitro* and *in vivo* environments, were evaluated using conventional techniques [measurement of antioxidant enzyme activities, total chlorophyll, TBARS and proline amounts, measurement of Na^+^ and K^+^ ion concentrations, and evaluation of gene expression by qPCR (*Ta*HKT2;1, *Ta*HKT1;5, *Ta*SOS1, *Ta*Na^+^/H^+^ vacuolar antiporter, *Ta*V-PPase, *Ta*V-ATPase, and *Ta*P5CS)] and RS. The hypothesis of this study is that RS has a high potential to shorten the process in breeding programs, which was tested by comparing RS with traditional techniques.

## 2 Materials and methods

### 2.1 Materials

**Figure 1:**
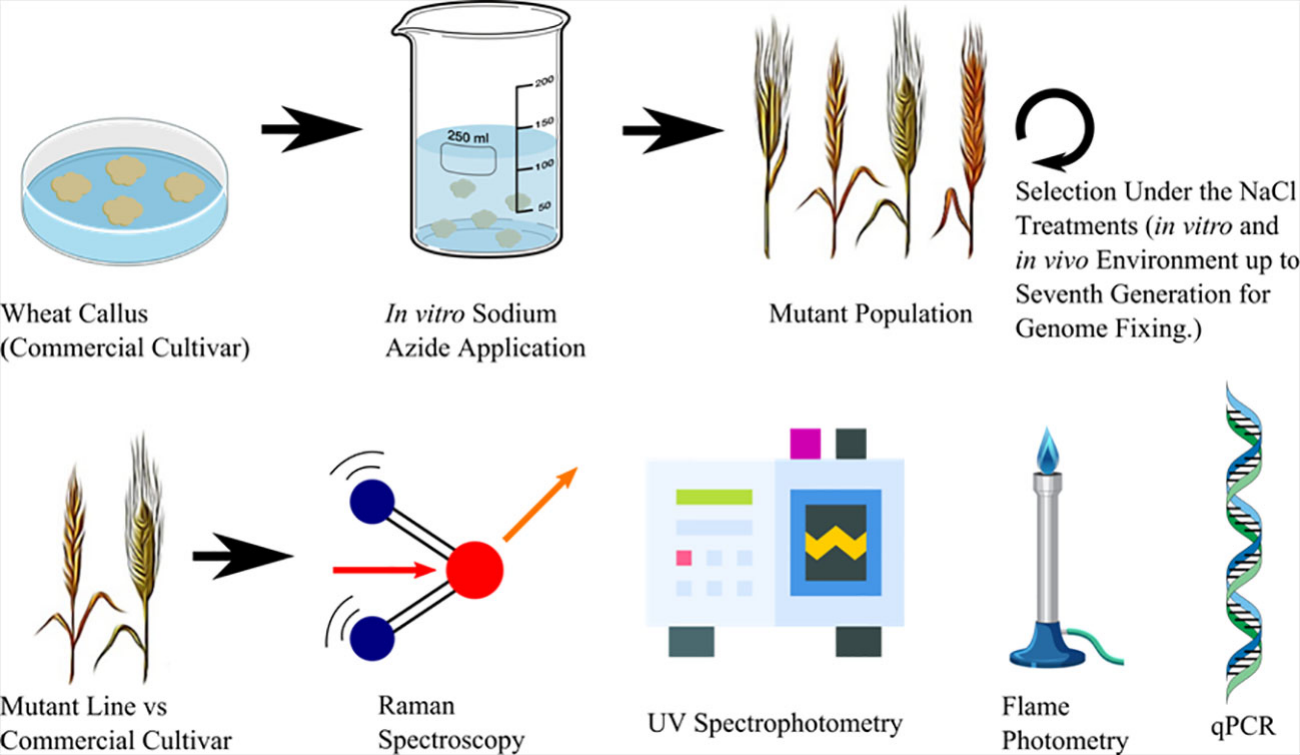
Outline of the experimental procedure for comparing conventional methods versus hightroughtput Raman base analysis to discriminate commercial wheat cultivar and advanced generation mutant wheat lines.

This study used the cultivar of bread wheat “Adana 99” (*T. aestivum* L. cv.), sourced from the “Eastern Mediterranean Agricultural Institute” in Adana, Turkey. Mutant lines were produced through 4 mM NaN_3_ treatment *in vitro*. Salt tolerance was assessed in treated and non-treated embryonic calli by exposing them to 125 mM NaCl in indirect regeneration media. The mutant lines were then segregated through several generations by using *in vitro* as well as *in vivo* techniques that involved exposing the plants to NaCl (Sen and Sarsu, 2018 and Sen and Sarsu, 2019). The outline of the experimental procedure of this study is given in **Figure 1**.

### 2.2 Methods

#### 2.2.1 Growth and stress treatments of materials

**Figure 2:**
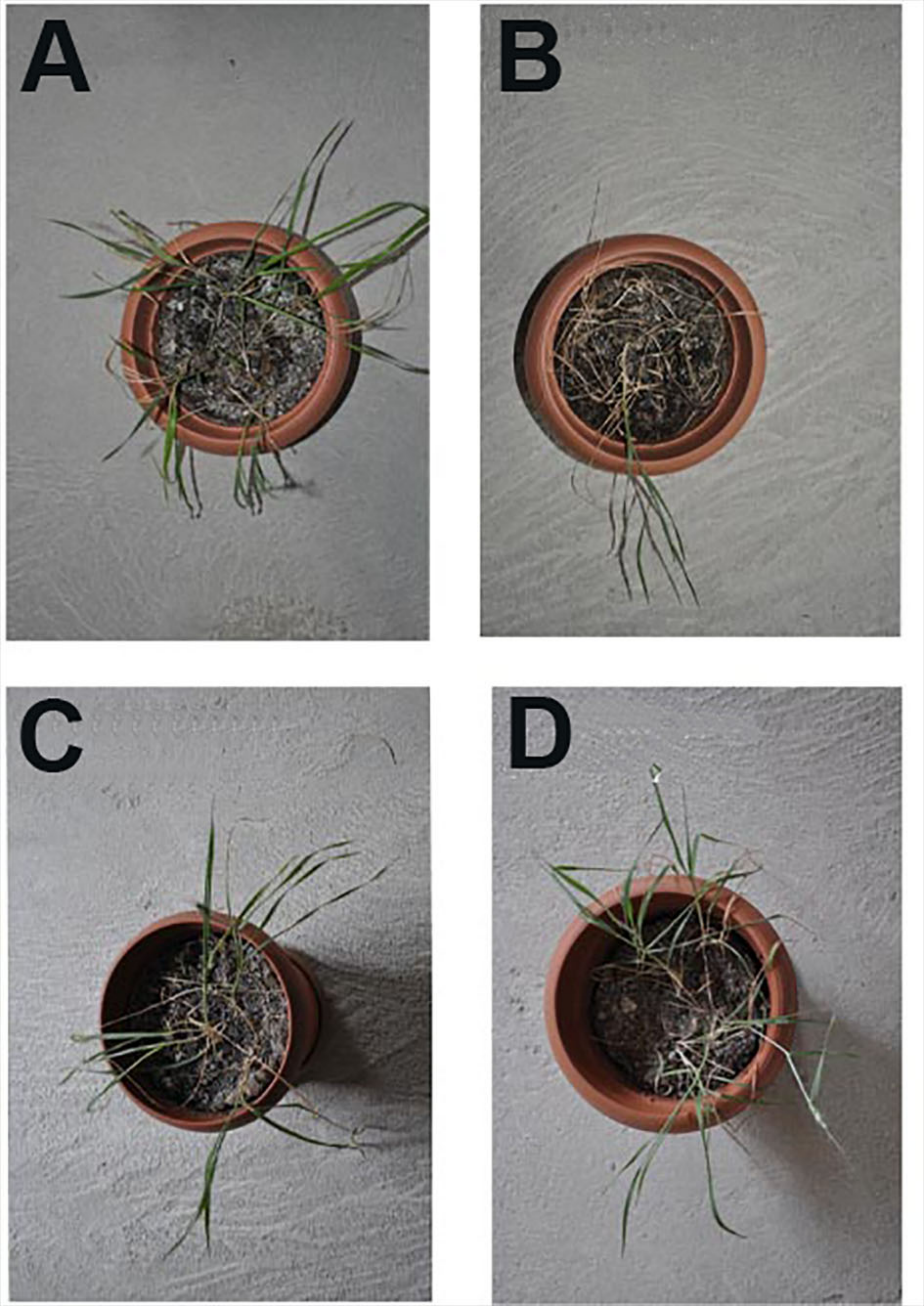
General view of commercial cultivar (as a control) and mutants under salt stress treatment. Control without salt stress treatments **(A)**, Control under 125 mM NaCl treatments **(B)**, Mutant Line 1 under 125 mM NaCl treatments **(C)** and Mutant Line 2 under 125 mMNaCl treatments **(D)**.

Both commercial cultivar and mutant line seeds were planted in pots filled with a soil mixture of peat moss and perlite in a 1:2 ratio (v/v). The pots were placed in a growth chamber with a 16-h light/8-h dark photoperiod, an irradiance of 500 μmol m^−2^ s^−1^, and a temperature of 26°C for 28 days. After 2 weeks of germination, irrigation was done on 14-day-old seedlings with a solution containing 1/10 Hoagland and 125 mM NaCl for 2 weeks, with a 3-day interval between waterings (**Figure 2**). Leaf samples were collected from the seventh-generation mutants and their parent plants (control) for biochemical and molecular analysis. For qPCR, harvesting of the plants was done at 0 and 2 h after treatment, and leaf samples were collected 28 days after treatment. These samples were stored at −80°C after freezing in liquid nitrogen for further procedure of RNA extraction.

#### 2.2.2 Measurement of antioxidant enzyme activities and proline content

Frozen leaf tissues (200 mg) were obtained through extraction buffer solution consisting of 100 mM phosphate buffer (pH 7.0), 1% polyvinylpyrrolidone 40 (PVP40) (Sigma-Aldrich GmbH, Germany) (w/v), and 0.1 mM disodium ethylenediaminetetraacetate dihydrate (Na_2_-EDTA) (Sigma-Aldrich GmbH, Germany) to measure the antioxidant enzyme’s activity. Centrifugation of samples was done at 13,000×*g* for 25 min at 4°C. The supernatant was utilized for further analysis. Bradford (1976) method was used to measure protein content. SOD activity was determined by monitoring the reduction of nitro blue tetrazolium chloride (NBT) (Sigma-Aldrich GmbH, Germany) induced by superoxide radical at 560 nm (Beauchamp and Fridovich, 1971). One unit of SOD activity was defined as the quantity of enzyme that causes a 50% inhibition of the photochemical reduction of NBT. The measurement of guaiacol peroxidase (POX EC. 1.11.1. 7) activity was performed at 470 nm by utilizing hydrogen peroxide (H_2_O_2_) (Sigma-Aldrich GmbH, Germany) and guaiacol (Sigma-Aldrich GmbH, Germany) as substrates. The decline of H_2_O_2_ was monitored at 240 nm for the determination of CAT activity (Aebi, 1984). For the determination of proline content, the ninhydrin method was applied (Bates et al., 1973).

#### 2.2.3 Measurement of chlorophyll and the end products of lipid peroxidation quantity

The chlorophyll quantification was done by using spectrophotometry, by following the method described by Arnon (1949). The lipid peroxidation end products were measured by determining the amount of TBARS using the TBA reaction method described by Heath and Packer (1968).

#### 2.2.4 Measurement of the cation concentration

The cations (sodium and potassium) were studied using atomic absorption flame emission photometry (AA-6501F; Shimadzu, Japan). The sample preparation was done using the method described by Xu et al. (2013).

#### 2.2.5 qPCR analysis

Specific primers were designed (listed in **Supplementary Table 1**) by using the internet-based tool Primer 3 (http://bioinfo.ut.ee/primer3-0.4.0/). To design these primers, the mRNA sequences for the target genes were obtained from the NCBI (National Center for Biotechnology Information) GenBank and loaded onto Primer 3. The default parameters for melting temperature (ranging between 60°C and 65°C), primer length (18–24 bp), and DNA fragment length (300–400 bp) were used. RNA was obtained from samples of frozen leaves by using TRIzol reagent, and cDNA was created using Superscript II reverse transcriptase following manufacturer instructions. Quantitative PCR (qPCR) was done on a Light Cycler Nano (Roche) machine by using SYBR Green Master Mix, and *Triticum aestivum* Actin (*Ta*Actin) and *Triticum aestivum* Glyceraldehyde-3-Phosphate Dehydrogenase (*Ta*GAPDH) genes were used as internal controls. A 25-μl PCR tube contains 300 ng of a cDNA template, 12.5 μl of Master Mix, and 0.4 μl of each primer (100 μM). The step-cycle program was as follows: 10 min at 95°C, followed by 45 cycles of 30 s at 95°C, and 1 min at 60°C. The relative gene expression was measured using the 2^−ΔΔCT^ equation (Livak and Schmittgen, 2001).

#### 2.2.6 Raman spectroscopy

RS measurements were taken in a custom system at Bogazici University Department of Physics. We built the system using three main parts: a diode laser (785 nm, 100 mW, LaserGlow), a spectrometer (QE-Pro, Ocean), and a microscope rigid-body microscope (Nikon Ti). The laser beam was cleaned up with a bandpass filter (Semrock), and the output beam was transmitted through a spatial filter to obtain a single longitudinal mode operation. The cleaned-up beam was sent to the first dichroic mirror (LP805, Thorlabs) and reflected to the other (SP 750, Thorlabs). The reflected beam from the last dichroic mirror was steered to the microscope objective (10×, 0.25 NA, Olympus). The backscattered beam (Rayleigh and Raman photons) was collected *via* the same light path with 180°C geometry. Finally, the Rayleigh photons were filtered at the first dichroic mirror. The Raman beam was sent to the focusing lens after transmission from a Raman edge filter (Semrock) for further Rayleigh filtering operation. The Raman beam was focused into the multimode fiber (0.22 NA, Thorlabs) using a fiber collimation package (Thorlabs). The coupled beam was sent to the spectrometer, and the spectra were visualized using the appropriate software (Oceanview).

### 2.3 Data analyses

Each experimental set consisted of up to 30 seedlings. To quantify the biochemical parameters and to perform qPCR, leaves were pooled randomly from three seedlings in each group. Statistical analysis was done by using one-way ANOVA (analysis of variance) on the spectrophotometric and gene expression data. Shapiro–Wilk test was used to check the data for normality, and differences between exposure groups were investigated using one-way ANOVA subsequently using Tukey’s *post-hoc* test. A *p*-value of less than 0.05 was considered statistically significant (Zar, 1984).

Raman spectral analysis was performed using the analysis codes we built on MATLAB, which is also open on GitHub (https://github.com/ikecoglu/PhotonicsLab). We applied spectral pre-processing on the raw spectra in three steps: Raman shift calibration, baseline correction, and normalization. Among these, we estimated the baseline using a polynomial curve fitting approach and then applied vector normalization (Heraud et al., 2006). To classify measurements, we first randomly split the data into 20%–80% test–train parts. Then, we trained a classification tree with Gini index as split criterion and a maximum of 100 splits, using the Classification Learner (MATLAB, 2021b).

## 3 Results

### 3.1 Measurement of antioxidant enzymes’ activity and the contents of proline, TBARS, and chlorophyll

**Figure 3:**
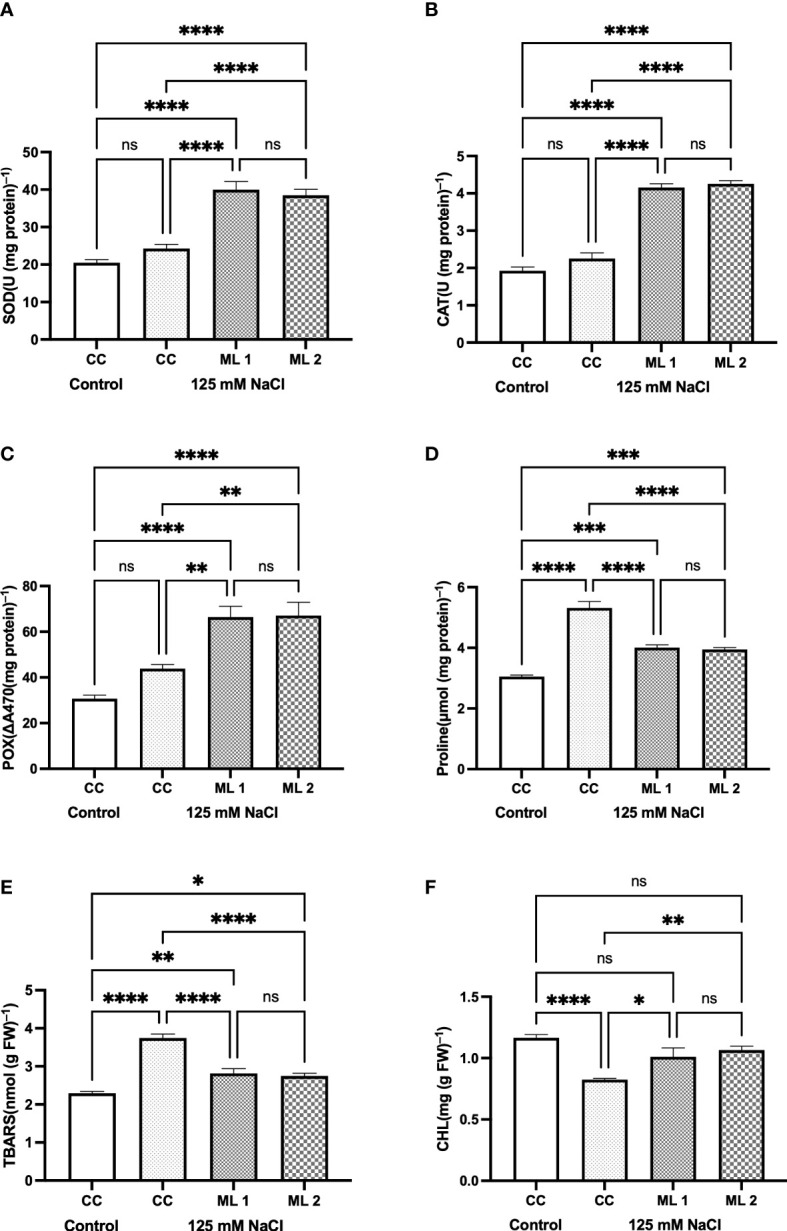
Effects of 125 mM NaCl stress on on SOD **(A)**, CAT **(B)**, POX **(C)**, Proline **(D)**, TBARS content **(E)**, and total chlorophyll **(F)** for the commercial cultivar and mutant lines. Significant differences were determined according to Tukey’s HSD test at *p < 0.05, **p < 0.01, ***p < 0.001, ****p < 0.0001, (ns, not significant; CC, Commercial cultivar; ML1, Mutant Line 1; ML2, Mutant Line 2). The values are means and standard errors for five replications of the experimental groups.

**Figure 3** illustrates the bioindicators of stress-related defense and impairment in the commercial cultivar (as a control), seedlings of the mutant lines for the control group, and those watered with 125 mM NaCl for 4 weeks. While on one hand there was no statistically significant difference between the mutant lines, and between the control and salt-stress-treated commercial cultivars in terms of SOD (**Figure 3A**), CAT (**Figure 3B**), and POX (**Figure 3C**) enzyme activities, on the other hand, a statistically significant difference was found between the control and stress-treated advanced mutant lines and also between the stress-treated commercial cultivar and advanced mutant lines. The increase in the enzyme activities of SOD, CAT, and POX in mutant lines was more pronounced than in other experimental groups. The mean increase in enzyme activities was measured as approximately two times more in mutant lines than the control. In the plants that were exposed to salt stress, proline content (**Figure 3D**) for mutant lines and the stress-treated commercial cultivar were statistically different compared to the control. TBARS content followed the same trend as that of proline (**Figure 3E**). TBARS (**Figure 3E**) content was also measured like proline. The total chlorophyll content shows no statistically significant difference between untreated (control) and salt-treated advanced generation mutant lines. However, the content of chlorophyll in the stress-treated commercial cultivars decreased as compared to control (*p* < 0.0001).

### 3.2 Measurement of cation concentrations

**Figure 4:**
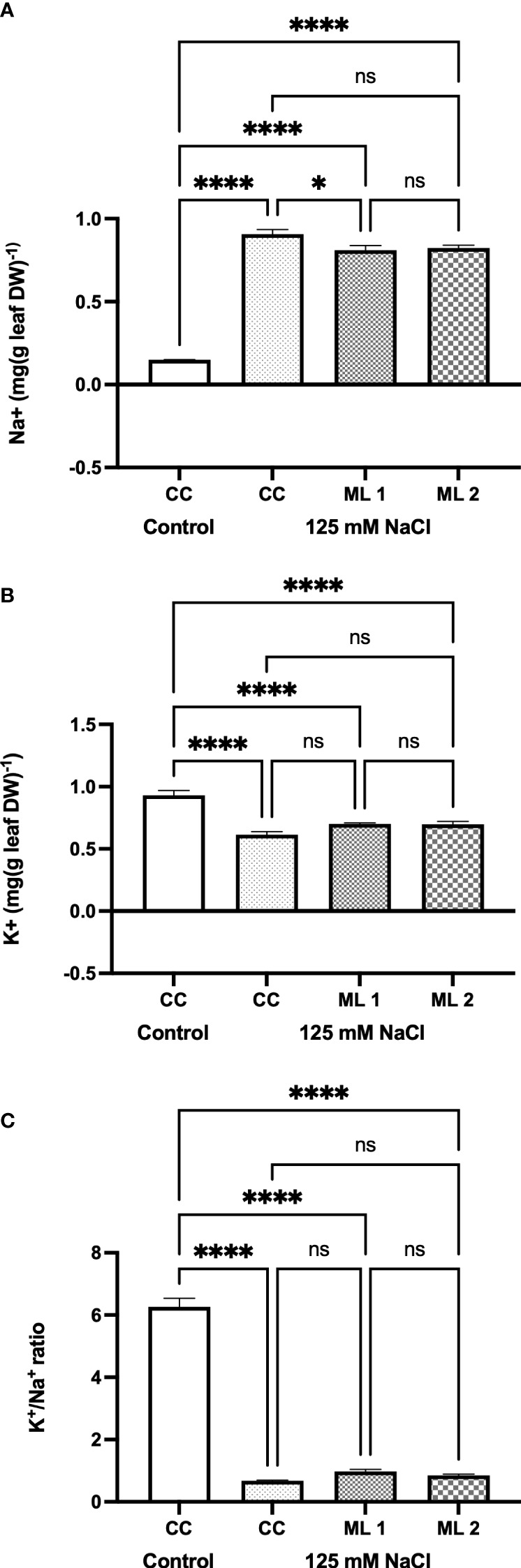
Effects of 125 mM NaCl stress on Na+ **(A)** and K+ **(B)** contents, and K+ / Na+ ratio **(C)** for the commercial cultivar and mutant lines. Significant differences were determined according to Tukey’s HSD test at *p < 0.05, ****p < 0.0001, (ns, not significant; CC, Commercial cultivar; ML1, Mutant Line 1; ML2, Mutant Line 2). The values are means and standard errors for five replications of the experimental groups.

Cation (Na^+^ and K^+^) concentration in control, commercial cultivars and advanced generation mutant lines under stress treatment is shown in **Figure 4**. The difference in the amounts of Na^+^ (**Figure 4A**) and K^+^ (**Figure 4B**) and the rate of K^+^/Na^+^ (**Figure 4C**) were statistically significant in the two advanced lines of mutants and the commercial cultivar under stress-treated conditions compared to control (*p* < 0.0001). While the content of Na^+^ in plants increased in the experimental groups exposed to stress in comparison to the control, the content of K^+^ and K^+^/Na^+^ ratio reduced in the same groups. However, there is no statistically significant difference between mutant lines and the salt-stress-treated commercial cultivar in terms of the amount of Na^+^ and K^+^, and the rate of K^+^/Na^+^ experimental parameters, but not in terms of the amount of Na^+^ ions between the salt-stress-treated commercial cultivar and Mutant Line 1.

### 3.3 Gene expression analysis

**Figure 5:**
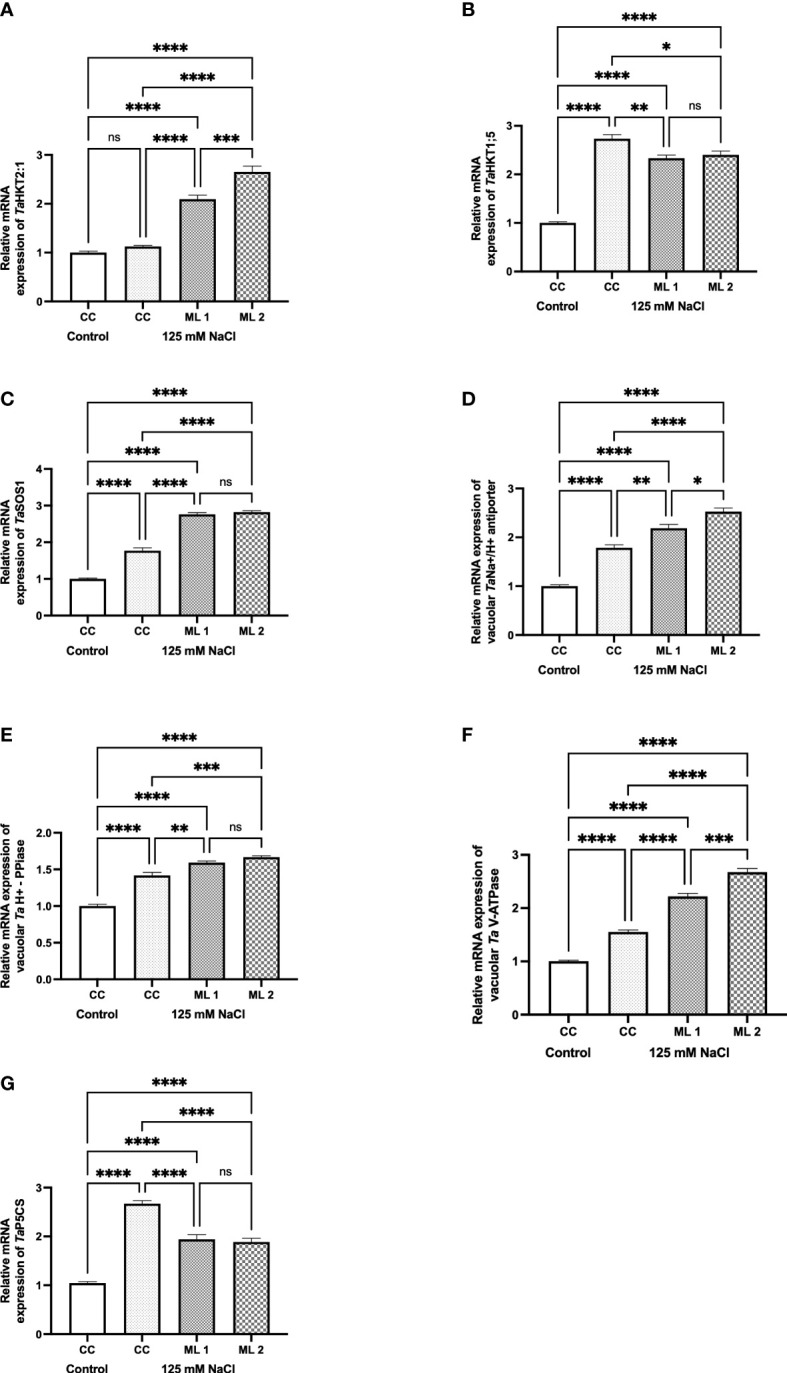
Effects of 125 mM NaCl stress on gene expression levels of *Ta*HKT2;1 **(A)**, *Ta*HKT1;5 **(B)**, *Ta*SOS1 **(C)**, *Ta*Na^+^/H^+^ vacuolar antiporter **(D)**, *Ta* vacuolar H^+^-PPase **(E)**, *Ta*vacuolarH^+^-ATPase **(F)**, and *Ta*P5CS **(G)** for the commercial cultivar and mutant lines. Significant differences were determined according to Tukey’s HSD test at *p<0.05, **p<0.01, ***p<0.001, ****p<0.0001 (ns, not significant; CC, Commercial cultivar; ML1, Mutant Line 1; ML2. Mutant Line 2). The values are means and standard errors for three replications of the experimental groups.

The genes *Ta*HKT2;1 (**Figure 5A**), *Ta*HKT1;5 (**Figure 5B**), *Ta*SOS1 (**Figure 5C**), *Ta*Na^+^/H^+^ vacuolar antiporter (**Figure 5D**), *Ta* vacuolar pyrophosphatase (**Figure 5E**), *Ta* vacuola*r* H^+^-ATPase (**Figure 5F**), and *Ta*P5CS (**Figure 5G**) are responsible for uptake and compartmentalization of intracellular K^+^ and Na^+^ content (**Figure 4**). When the stress-treated commercial cultivar and advanced mutant lines and the non-stressed commercial cultivar were compared in terms of gene expression, it was determined that there were statistical differences at various levels. Except for the stress applied to the commercial cultivar in *Ta*HKT2;1, gene expression increased to varying degrees in all stress-exposed groups. In fact, the increase in *Ta*HKT1;5 and *Ta*P5CS gene expressions in the stress-treated commercial cultivar was statistically higher than the advanced mutant lines.

### 3.4 Raman spectroscopy

**Figure 6:**
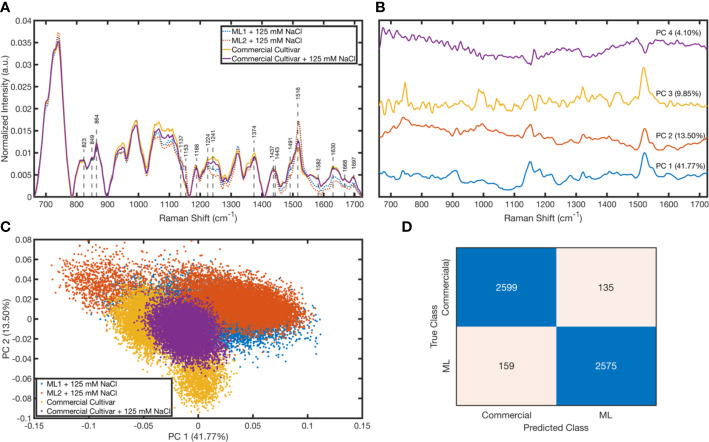
Raman spectroscopy results: Mean normalized spectra for each cultivar **(A)**. Loading plots for the first four principal components **(B)**. Scattering of cultivars in the PC 1–PC 2 space **(C)**. Confusion matrix of the tree classifier with the test data **(D)**.

The three different leaves for the commercial cultivar were scanned, i.e., commercial cultivar with 125 mM salt and two mutant lines with 125 mM salt. In each scan, 4,221 spectra were measured; thus, a total of 12,663 spectra were taken for each class. To eliminate outliers, a quantile elimination was utilized, which left only the 0.005–0.995 interval. After elimination, the classes had differing numbers of spectra to prevent class imbalance random sampling, and a reduced class size of 6,836 was used, which was the size of the smallest group after elimination. Therefore, after the preprocessing, we had 13,672 measurements from the commercial cultivar (6,836 from controls and 6,836 from the salt-treated group) and 13,672 from mutant lines (6,836 from Line 1 and 6,836 from Line 2). In **Figure 6A**, the average baseline-corrected and normalized Raman spectra taken from commercial cultivars and mutant lines is shown. Some particular Raman shift values whose corresponding assignments can be seen in **Table 1** were marked. It was found that the commercial cultivar measurements differentiate in terms of the peaks around 1,224, 1,241, 1,374, and 1,516 cm^−1^ when they are treated with salt. On the other hand, it is demonstrated in **Figure 6A** that the peaks around 1,241, 1,582, and 1,630 cm^−1^ corresponding to mutant line measurements change in intensity when they are treated with salt. There is a considerable separation between commercial and mutant lines at 1,153, 1,241, 1,491, 1,516, 1,582, 1,630, 1,668, and 1,697 cm^−1^.

**Table 1:** Tentative band assignments for the changing peak wavenumbers.

Peak	Change (from com to ml, shift, and/or intensity)	Assignment	Reference
823	Decrease	γ(CO_2_), histidine solid, isoleucine solid, valine solid/equatorial anomeric H (a-anomers and a-glycosides)	(Synytsya et al., 2003; Zhu et al., 2011)
849	850 - decrease	Cγ-twist, Cϵ-wag, Cβ-wag, lysine solution, tryptophan solid/Me-Pec	(Synytsya et al., 2003; Zhu et al., 2011)
864	Decrease	γ(CH), gallic acid	(Espina et al., 2022)
1,137	Increase	C–N and C–O stretching, valine solution	(Fasman et al., 1978; Zhu et al., 2011)
1,153	1,150 - increase	Skeletal C–O and C–C vibration, glycosidic bonds and pyranoid ring, acetylated pectates	(Synytsya et al., 2003)
1,188	1,185 - increase	ν(C-O-H) next to aromatic ring + σ (CH), carotenoids/phenylalanine solid	(Zhu et al., 2011; Morey et al., 2021)
1,224	1,222	ν(C–N) + C–C + δ(C–H), histidine solid	(Mesu et al., 2005; Zhu et al., 2011)
1,241	Decrease	δ(CH), ν(ring), ν(CO), pyrogallol	(Espina et al., 2022)
1,374	1,377	dsym(CH3(CO)), 1,8-cineol	(Schulz and Baransca, 2007)
1,437	1,434	d(CH2), a-bisabolol/tyrosine solid	(Schulz and Baransca, 2007; Zhu et al., 2011)
1,443	Decrease	ν(ring), δ(OH), gallic acid/arginine solid, histidine solution	(Zhu et al., 2011; Espina et al., 2022)
1,491	Decrease	Aromatic ring stretch, asymmetric, lignin	(Agarwal and Reiner, 2009)
1,516	1,519 - increase	ν(ring), δ(C–OH), δ(CH)/β-carotene/glycine solid	(Zhu et al., 2011; Jehlička et al., 2014; Espina et al., 2022)
1,582	Decrease	Stretching vibrations of CO_2_^−^, isoleucine solid, leucine solid	(Zhu et al., 2011)
1,630	Decrease	Aromatic and aliphatic –C=C–, C=O stretching, benzene ring/serine solid	(Schulz and Baransca, 2007; Zhu et al., 2011)
1,668	Decrease	ν(0,0), collagen solid, amide-I	(Fermandjian et al., 1972; Zhu et al., 2011)
1,697	Decrease	CO stretching	(Schulz and Baransca, 2007)

To explore how the measurements are differentiated from each other, we performed a principal component analysis (PCA). We plotted its loadings (**Figure 6B**) and scores (**Figure 6C**). The peaks in the loading plot determine the peaks with high variations among the sample, which give a solid output to trace the varying chemical bonds between the classes. According to the loading plot, the peaks around 739, 910, 1,150, 1,182, 1,281, 1,322, and 1,522 cm^−1^ have high variation, also visible in **Figure 6A** as intensity differences between groups. Moreover, **Figure 6C** shows that even with just PC 1 and PC 2 (55.27% explained variance in total), the commercial cultivar and mutant lines separate from each other. Also, the salt-treated and untreated commercial cultivars have slight differences, which is not apparent for the mutant lines that have the same medium salt concentration. To quantitatively observe how well the commercial cultivar and mutant lines separate from each regardless of their salt concentration, a tree classifier was plotted using the randomly selected 80% of the data. **Figure 6D** shows that the confusion table of this model applied to the remaining 20% of the test set. According to this table, we could predict these classes with 94.62% accuracy. Our sensitivity and specificity were 95.06% and 94.18%, respectively.

## 4 Discussion

In this study, random mutations were created in the wheat genome by sodium-azide application, in order to elevate the tolerance of the wheat plant to salinity stress, which has a significant place in human diet, in addition to the fact that the salt tolerance of the obtained genotypes was followed up to the seventh generation by being tested in *in vitro* and *in vivo* environments. With the study carried out in the seventh generation, their resistance to salt was also revealed. Biochemical analysis based on a UV spectrophotometer and qPCR analysis based on the expression of genes that are related to the uptake and compartmentalization of Na^+^ into the cell, which is used frequently nowadays as a conventional method for the differentiation of advanced generation mutant lines and the commercial wheat cultivar from which they are derived, were examined. In recent years, this analysis technique has been used to see if RS, which is one of the modern techniques with high output, can make this distinction in a shorter time.

Salt stress can have a significant impact on a plant’s physiological and metabolic processes, leading to reduced growth and development. The extent of these effects depends on the duration and severity of the stress (Gupta and Huang, 2014). Salt stress or salinity triggers osmotic stress by preventing the plant from absorbing water from the soil in the initial phase, which can take minutes to days. By increasing the production of ROS, osmotic stress causes processes such as damage to cell membranes, denaturation of proteins, lesions on DNA, imbalance in nutrient absorption, and decreased photosynthetic activity (Gupta and Huang, 2014; Hanin et al., 2016). To reduce these negative effects, plants have developed various defense systems. The increase in activity due to stress in antioxidant enzymes such as SOD, POX, and CAT is one of these systems. In this context, having a developed defense system in plants has emerged as a frequently preferred breeding criterion in recent years (Chen et al., 2011; Song et al., 2012; Gupta and Huang, 2014; Hanin et al., 2016; Wang et al., 2019). SOD is a metalloenzyme that forms hydrogen peroxide by reducing the superoxide anion. CAT and POX are enzymes responsible for the detoxification of hydrogen peroxide formed in various compartments within the cell (Gill et al., 2013; Gupta and Huang, 2014; Hanin et al., 2016). In the current study, no significant difference in the increase of SOD, CAT, and POX antioxidant enzyme activities between the salt-treated and untreated experimental groups of the commercial cultivar was observed, while on the other hand, there was a significant increase in salt-treated advanced generation mutant lines as compared to control and the salt-treated commercial cultivar. Depending on the salt stress application of the advanced generation mutant lines, no such difference was detected in the comparison between them. Proline, an osmoprotectant, helps maintain cell membrane stability under stress conditions. Genotypes that are resistant to stress are expected to accumulate higher levels of proline under stress conditions, and proline accumulation is often used as a selection criterion in breeding programs (Khan et al., 2009). In the current study, it showed a statistically significant increase in all experimental groups compared to the untreated commercial cultivar. This increase was highest in the commercial cultivar, which was subjected to salt stress. Maintaining the stability of cell membranes and low degradation of chlorophyll are among the biochemical parameters used in breeding programs today (Song et al., 2012). Increased ROS production in the cell due to stress conditions causes detrimental effects on the stability of cell membranes and macromolecules such as chlorophyll, and cellular functions are interrupted as a result (Gupta and Huang, 2014). This effect was found to be less in genotypes with high stress tolerance. In our study, the content of TBARS, which was measured as a bioindicator of cell membrane damage, increased in the salt-stressed groups compared to the control. While this increase was determined in the highest content of salt stress application in the commercial cultivar, the increase in the advanced generation mutant lines was statistically significant, but it was less than the salt-stressed commercial cultivar. The degradation in the content of chlorophyll was lower in the salt-stressed advanced generation mutant lines than in the salt-treated commercial cultivar. Like our study, increased activity of CAT, SOD, and POX enzymes, which are indicators of stress tolerance, was observed in transgenic potato lines that had been genetically modified to overexpress the *At*HKT1 gene and were exposed to salt stress. This suggests that *At*HKT1 gene transfer may improve salt tolerance in potato plants (Wang et al., 2019). In this study, although proline increased in the salt-stressed commercial cultivar compared to mutant lines, the activities of SOD, CAT, and POX increased more in mutant lines than in the commercial cultivars that were subjected to salt stress. This explains why the amount of TBARS resulted in less increase and less degradation of chlorophyll in mutant lines.

Salinity also causes hyper-ionic stress. In this context, the most important effect is the increase in the absorption of Na^+^ and Cl^−^ from the soil *via* plant roots. The influx of these ions into the cell leads to ionic imbalance. Increasing Na^+^ ion uptake triggers physiological and metabolic imbalances in plant cells by decreasing K^+^ ion absorption, which is a crucial element for plant growth. In our study, Na^+^ absorption increased in all experimental groups to which salt stress was applied, compared to the control group to which salt stress was not applied (**Figure 4**). Therefore, the K^+^/Na^+^ ratio has been accepted as an important selection criterion in plant breeding in recent years. In order to maintain this ratio, plants have developed various mechanisms such as compartmentalizing the absorbed Na^+^ ion in vacuoles (Benderradji et al., 2011). Plants play a role in Na^+^ entry from the soil to the plant with the Na^+/^H^+^ antiporter sited in the SOS1 plasma membrane. The HKT gene family controls K^+^ transport across the plasma membrane into the cell. While HKT1 is sensitive to Na^+^, HKT2 is responsible for the uptake of both Na^+^ and K^+^ into the cell. In our study, the transcriptional activity of HKT2;1 was found to be higher in mutant lines exposed to salt stress than in the control group exposed to and not exposed to salt stress. Although the activity of HKT1;5 was higher in the control group exposed to salt stress, the mutant lines subjected to salt stress were also found to be higher than the control in terms of the transcriptional activity of HKT1;5. SOS1 was high in all experimental groups exposed to salt stress (**Figure 5**). The increase in the transcriptional activity of these three genes indicates that the uptake of Na^+^ from the soil to the plant is high. As a matter of fact, the Na^+^ ion contents measured in our study also confirm this situation (**Figure 3**). A durum wheat study found that overexpression of the sodium-hydrogen ion (Na^+^/H^+^) antiporter SOS1 gene in transgenic plants of *Arabidopsis* caused increased salt tolerance (Feki et al., 2014). In his study, Zhang et al. (2017) demonstrated that the synergistic regulation of HKT1;5, SOS1, and NHX1 transcription activities was useful in maintaining homeostasis of Na^+^ in the *Puccinellia tenuiflora*, which is a halophyte plant, in both high- and low-salt-stress conditions. Although the plant absorbs such a high amount of Na^+^ from the soil, we examine how the control group and mutant lines exposed to salt stress tolerate this accumulation, and how the vacuolar Na^+^/H^+^ antiporter, V-ATPase (vacuolar type H^+^-ATPase), and V-PPase (vacuolar pyrophosphatase) pump increased in the experimental groups exposed to stress compared to the control group that was not exposed to stress (Gupta and Huang, 2014). This increase occurred in higher amount in the stressed mutant lines than in the control group (**Figure 5**). In a study conducted with *Suaeda sake* on halophyte, it was determined that V-ATPase activity increased under salinity stress conditions, while V-PPase did not play a major role (Wang et al., 2001). Brini et al. (2007) found that overexpression of the wheat Na^+^/H^+^ antiporter TNHX1 and H^+^-pyrophosphatase TVP1 genes in *Arabidopsis thaliana* plants improved their tolerance to both salt and drought stress. Overexpression of the vacuolar Na^+^/H^+^ antiporter and H^+^-pyrophosphatase pump (H^+^-PPase) has also been shown to increase plant tolerance to salinity and drought (reviewed by Brini et al., 2007). Xue et al. (2004) found that increasing the level of the vacuolar Na^+^/H^+^ antiporter in wheat plants improved their tolerance to salt stress and increased grain yield in saline soils. In the current study, despite the transcription activities of the genes involved in the uptake of Na^+^ from the soil to the plant and vacuolar transport of Na^+^ in plant cells, the activities of the intracellular defense parameters SOD, CAT, and POX were high in mutant lines, and the intracellular damage parameters TBARS and chlorophyll were high in mutant lines and have a lower value than the control group exposed to stress. In addition to being an osmoprotectant in the cell, proline plays many important roles in maintaining the integrity of cell membranes, as an intracellular signal molecule, as a free radical scavenger, as a chaperone, and as a chelator (Hayat et al., 2012). The P5CS gene is responsible for the transcription of proline (Gupta and Huang, 2014). Proline is compatible with the accumulation of intracellular proline under stress in experimental groups. Gene expression and proline accumulation were higher in all stress-exposed experimental groups than in the non-stressed control group. In this study, the highest values of both parameters measured for proline were measured in the control group exposed to stress. However, the contents of TBARS and chlorophyll, which are frequently used as injury parameters, show that the damage is also high in the control group exposed to stress. Proline is an important molecule that acts as an intracellular osmoprotectant (Song et al., 2012). It is clear from the results that proline content alone is not sufficient to keep the effects of damage under control. In the light of recent studies, tolerance to salt stress in plants has been reported as a complex genetic event in which many mechanisms are involved. In this context, studies show that multiple genes interact with each other in additive, dominance, and reciprocal interactions at the genetic level. In the light of this information, it is important to note that there are likely many other factors that contribute to the salt stress tolerance of mutant lines beyond the parameters that were measured in this study. However, making these evaluations with conventional methods leads to loss of time, increase in workforce, and cost increase. In recent years, researchers have started to use new techniques to eliminate these disadvantages. One of these techniques is RS. In this context, we tested whether the distinction between mutant lines and control groups under stress conditions can be made using RS, similar to conventional techniques, using RS in our study.

We show in **Figure 6A** that the Raman spectra of the commercial cultivar were different from the mutant lines. This difference was quantified using multivariate statistical analysis methods, and we found that commercial cultivar responses were significantly different from mutant lines. In contrast, differences between commercial groups with different salt concentrations were not significant. Raman spectral data analysis demonstrated high sensitivity and specificity (94.18% and 95.06%) to separate two mutant line types from each other. The differences in peaks show that the protein-related bands in mutant lines have a general decreasing trend in intensity when compared to the commercial cultivar. Amide-I (1,630 and 1,668 cm^−1^), Histidine, Lysine, Arginine, and Leucine bands (823, 849, 1,241, 1,443, and 1,582 cm^−1^) are the decreasing wavenumbers as shown in **Table 1**. Beta-carotene peaks at 1,153 and 1,519 cm^−1^ showed increasing trends when the normalized Raman intensities of the two mutant lines were compared. Amino acids, for example, histidine, leucine, isoleucine, and arginine, are significant amino acids that are found freely in the cell and participate in the structure of proteins. These play important roles in maintaining the continuity of intracellular metabolism and in the intracellular defense system. Similar to our study, Farhangi-Abriz and Ghassemi-Golezani (2016) showed that these amino acids decreased under stress conditions. Carotenoids are a light-trapping pigment in photosynthetic organisms and one of the structural components of photosynthesis. Carotenoids also act as scavengers of superoxide anions in the photosynthetic apparatus. Therefore, in our study, the increase in the carotenoid content in the aqueous phase shows that they play a vital part in protection against stress. In the light of all these measurements, it is once again understood how complex the defense system is in plants.

In conclusion, we have seen that RS has potential in plant breeding programs by eliminating the disadvantages of conventional methods in the discrimination of the breeding material or the new genotype from the source from which it was obtained.

## Data availability statement

The spectroscopy data and the trained models that support the findings of this study are openly available in Zenodo at doi: 10.5281/zenodo.7644520.

## Author contributions

AS constructed mutant populations and obtained mutant lines used in this study, planned and performed experimental design and data analysis of biochemical parameters and qPCR analysis. MA performed the experiments of biochemical analysis. UP, and IK planned and performed experimental design and data analysis of the Raman spectroscopy experiments. MBU supervised the Raman spectroscopy experiments and found funding for research. All authors reviewed the manuscript.
