# Supplementary material for: Differentiation of advanced generation mutant wheat lines: Conventional techniques versus Raman spectroscopy
Source: Front Plant Sci. 2023 Feb 23;14:1116876. doi: 10.3389/fpls.2023.1116876 (PMC9997642; doi:10.3389/fpls.2023.1116876)
Supplement: Supplementary file 1 [file Table_1.docx]

**Supplementary Table 1.** Designed probes for detection of gene expression levels in the wheat genome.

| **Genes** | **GenBank Accession Nos** | **Amplicon  Sizes (base pair)** | **Primers** |
| --- | --- | --- | --- |
| *Ta*HKT1;5 | JQ677812.3 | 184 | F: 5’-TGATGCTGGTCTACCTGAGC-3' |
|  |  |  | R: 5’-GGCATGACTAGGGCAGGAG-3' |
| *Ta*HKT2;1 | HF937364.1 | 109 | F: 5'- GGTACTTGCGTGATCCAT-3 |
|  |  |  | R: 5'- AGCACGATGTTGATCCCTTT-3' |
| *Ta*Na^+^/H^+^ vacuolar antiporter | JQ180507.1 | 158 | F: 5’- ATTCTTCACCAGCACCGTTC-3’ |
|  |  |  | R: 5’- TCAGATCCAGCAGCATTGAC-3’ |
| *Ta*vacuolar pyrophosphatase | AY296911.1 | 190 | F:5'TGATATTGCTGGGATGGGATCAG-3' |
|  |  |  | R:5'AAAGAAATCAGTCGCAAACAGGG-3' |
| *Ta*vacuola*r* H^+^-ATPase | EV254356.1 | 200 | F: 5'-GTCTGTGTGCAGACTCTGGT-3' |
|  |  |  | R:5'-GACCTCGCCTGTGAACTGAA-3' |
| *Ta*SOS1 | KF169799.1 | 109 | F: 5’-AGAGGGGCGAAGGAATAAAA-3’ |
|  |  |  | R: 5’-AAAAATGCGCACTCCCATAC-3' |
| *Ta*P5CS | AY888045.1 | 157 | F: 5'TACAGTTCTATGGCTTGCACAGT-3' |
|  |  |  | R:5'ATACGGCAGCACTATCAACTTGA-3' |
| *TaGAPDH* | AF251217.1 | 175 | F:5'-GGAGGAGTCTGAGGGAAACC-3’ |
|  |  |  | R:5'-GCTGTATCCCCACTCGTTGT-3' |
| *TaActin* | GQ339780.1 | 208 | F:5’-CGTGTTGGATTCTGGTGATG-3’ |
|  |  |  | R: 5’-AGCCACATATGCGAGCTTCT-3’ |
